# Association Between Pre-Pregnancy Body Mass Index and Miscarriage in an Assisted Reproductive Technology Population: A 10-Year Cohort Study

**DOI:** 10.3389/fendo.2021.646162

**Published:** 2021-06-16

**Authors:** Pengfei Qu, Mingxin Yan, Doudou Zhao, Dongyang Wang, Shaonong Dang, Wenhao Shi, Juanzi Shi, Chunli Zhang

**Affiliations:** ^1^ Translational Medicine Center, Northwest Women’s and Children’s Hospital, Xi’an, China; ^2^ Departments of Pediatrics and Neonatology, Children’s Hospital of Fudan University, Shanghai, China; ^3^ Department of Epidemiology and Health Statistics, School of Public Health, Xi’an Jiaotong University Health Science Center, Xi’an, China; ^4^ Assisted Reproduction Center, Northwest Women’s and Children’s Hospital, Xi’an, China; ^5^ Department of Obstetrics, Northwest Women’s and Children’s Hospital, Xi’an, China

**Keywords:** body mass index, obesity, miscarriage, assisted reproductive technology, twin pregnancy

## Abstract

**Objective:**

To investigate the association between pre-pregnancy body mass index (BMI) and miscarriages in women who required assisted reproductive technology (ART) for conception.

**Methods:**

A retrospective cohort study was conducted using a 10-year (2006–2015) sample of 14,994 pregnancy cycles with ART treatment in Northwest Women’s and Children’s Hospital, Xi’an, China. The effects of women’s BMI before pregnancy on early miscarriage and miscarriage were assessed using generalized estimating equation models.

**Results:**

The risks of early miscarriage and miscarriage were higher in the obese group than in the normal weight group [early miscarriage: relative risk (RR) = 1.36, confidence interval (CI): 1.12–1.65; miscarriage: RR = 1.40, 95% CI: 1.17–1.68]. Pre-pregnancy underweight was not associated with an increased risk of early miscarriage or miscarriage. We observed interactions between pre-pregnancy BMI and singleton or twin pregnancy in early miscarriage and miscarriage (P = 0.017 and P = 0.003, respectively). Twin pregnancy increased the effects of pre-pregnancy BMI on early miscarriage and miscarriage (early miscarriage: a. singleton pregnancy: RR = 1.02, 95% CI: 1.01–1.04; b. twin pregnancy: RR = 1.08, 95% CI: 1.03–1.13; miscarriage: a. singleton pregnancy: RR = 1.02, 95% CI: 1.01–1.04; b. twin pregnancy: RR = 1.08, 95% CI: 1.05–1.13).

**Conclusions:**

Pre-pregnancy obesity was associated with higher risks of early miscarriage and miscarriage in the ART population, and twin pregnancy increased the effects of pre-pregnancy BMI on early miscarriage and miscarriage. Women should maintain a normal BMI before ART initiation to prevent adverse pregnancy outcomes.

## Introduction

In the past 40 years, assisted reproductive technologies (ART) have become a common option for infertile couples worldwide (1). It is estimated that eight million individuals have been conceived using ART (2). Compared with spontaneous conception, ART is associated with an increased risk of multiple gestations, miscarriage, congenital malformations, and preterm birth (3–6).

The prevalence of overweight and obesity has increased in an alarming way in the past 50 years worldwide, and more than 39% of the adult population worldwide is now classified as overweight or obese (7, 8). With its economic development, diet, and lifestyle change, the prevalence of overweight and obesity in China is gradually reaching that in developed countries (9, 10). The prevalence of overweight or obesity in adults has increased from 22.8% to 30.1% between 2002 and 2012 in China (11). Pre-pregnancy BMI is an important indicator of pregnancy outcomes (12, 13). Nevertheless, studies on the associations between pre-pregnancy BMI and miscarriages are limited, and the conclusions are inconsistent for pregnant women receiving ART treatment (14–18). Although some studies have reported that pre-pregnancy underweight and obesity both increase the miscarriage rate of pregnant women receiving ART treatment (19, 20), other studies have reported no adverse effects of pre-pregnancy underweight, overweight, or obesity on the miscarriage rate of ART (14, 18). Thus, the objective of the present study was to analyze the influence of pre-pregnancy BMI on miscarriage in women receiving ART treatment. In total, 10 years of patient records, including demographic characteristics, ART treatments, and pregnancy outcomes, were collected and used to compare early miscarriage and miscarriage among different BMI groups in a single ART center in Xi’an, Shaanxi Province, Northwest China.

## Materials and Methods

### Study Design and Population

**Figure 1:**
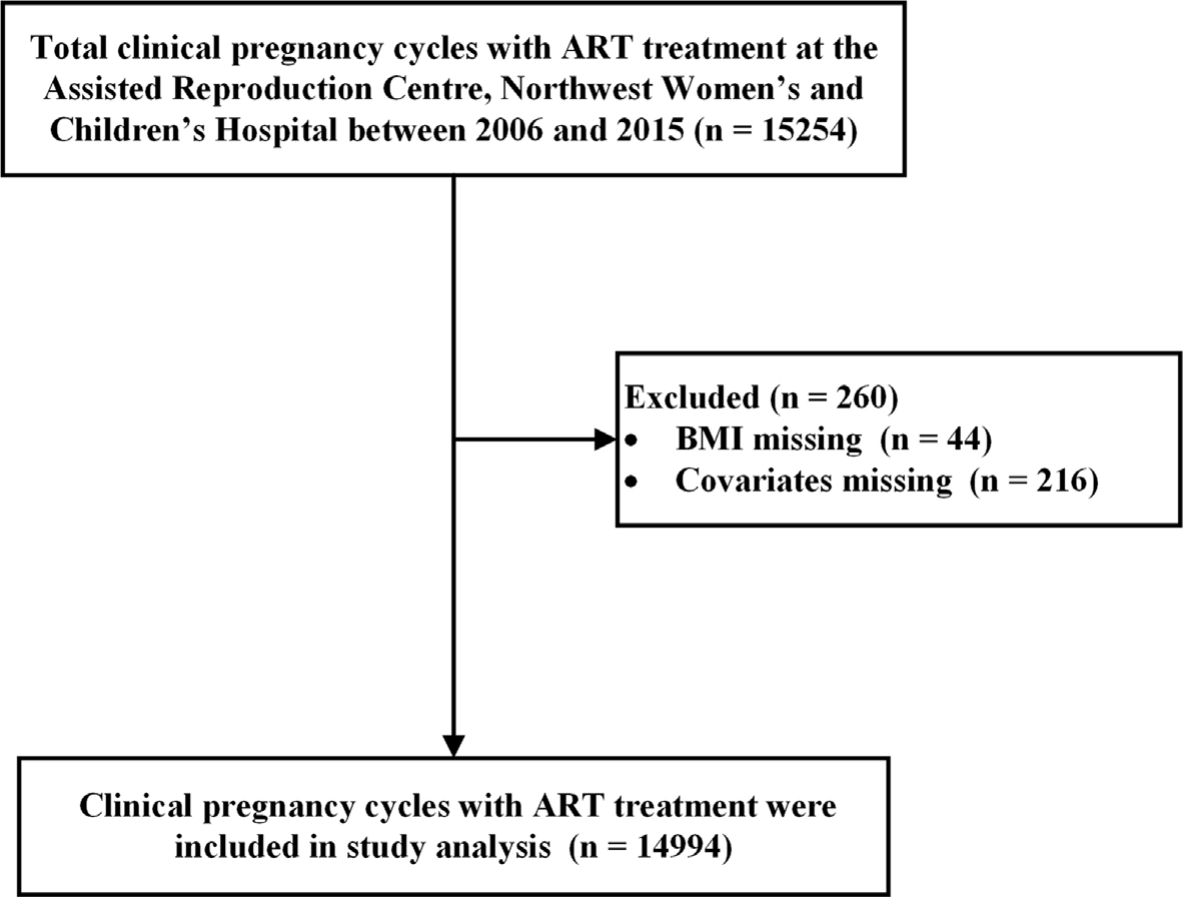
Eligibility assessment with exclusion criteria.

A retrospective cohort study was conducted using 10 years of clinical data (2006–2015) from the ART center at Northwest Women’s and Children’s Hospital, Northwest China. A total of 15,254 pregnancy cycles were conceived with *in vitro* fertilization (IVF)/intracytoplasmic sperm injection (ICSI) treatment. Consequently, 44 pregnancy cycles with missing pre-pregnancy BMI and 216 pregnancy cycles with missing covariates were excluded, leaving a total of 14,994 pregnancy cycles enrolled in this study (**Figure 1**).

Shaanxi Province requires that pregnancy outcomes (including early miscarriage and miscarriage) be reported in the Shaanxi Assisted Reproduction Database. In this study, all pregnancy outcomes were collected from the Assisted Reproduction Database. Demographic data and ART treatment data were collected and assessed by each patient’s clinician.

### BMI Assessment

Weight (nearest 100 g) and height (nearest 1 cm) were measured and recorded by a trained nurse one month before ART treatment. BMI was calculated as weight/height^2^ (kg/m^2^). Based on the Chinese criteria (21), pregnant women were divided into four BMI groups: underweight (BMI < 18.5 kg/m^2^), normal weight (18.5 ≤ BMI < 24.00 kg/m^2^), overweight (24 ≤ BMI < 28.00 kg/m^2^), and obese (BMI ≥ 28 kg/m^2^). Based on the World Health Organization (WHO) criteria (22), pregnant women were divided into four BMI groups: underweight (BMI < 18.5 kg/m^2^), normal weight (18.5 ≤ BMI < 25.00 kg/m^2^), overweight (25 ≤ BMI < 30.00 kg/m^2^), and obese (BMI ≥ 30 kg/m^2^).

### Definitions of Pregnancy Outcomes

Clinical pregnancy was confirmed using ultrasound at 26 days after blastocyst transfer or 28 days after cleavage-stage embryo transfer. Singleton and twin pregnancies were confirmed using ultrasonographic visualization of the gestational sacs and fetal heart. Early miscarriage and miscarriage were the primary outcome measures in this study. Early miscarriage was defined as the loss of pregnancy before 12 weeks of gestation. Miscarriage was defined as the loss of pregnancy before 20 weeks of gestation.

### Confounding Variables

Based on the literature (23), potential factors correlated to pregnancy outcomes, such as patient baseline demographic characteristics, clinical characteristics, and treatment procedure, were also collected for the study participants. These included maternal age, smoking history (yes or no), gravidity (0, 1–2 and ≥3), parity (0 or ≥1), main etiology of infertility (tubal factor, ovarian factor, male factor, and other reasons), year of transfer (2006–2009, 2010–2012, and 2013–2015), sperm donation (yes or no), fertilization method (ICSI, IVF, and IVF + ICSI), fresh/frozen embryo transfer, blastocyst/cleavage-stage transfer, assisted hatching (yes or no), antral follicle count, basal serum follicle stimulating hormone (FSH) level, endometrial thickness, number of embryos transferred (1, 2, or ≥3), and singleton or twin pregnancy.

### Statistical Analysis

The participants’ baseline characteristics are summarized using mean and standard deviation for normally distributed continuous variables, median and interquartile range (IQR) for non-normally distributed continuous variables, and counts and proportions for categorical variables. The chi-squared test or Fisher’s exact test was performed to compare categorical variables. Analysis of covariance and the Kruskal–Wallis test were performed to compare normally distributed variables and non-normally distributed variables, respectively.

Generalized estimating equation (GEE) models with log link were employed to analyze the relationships between pre-pregnancy BMI and miscarriages, and in the same woman as a cluster effect. All multivariate analyses were adjusted for all baseline covariates (age, smoking history, gravidity, parity, main etiology of infertility, year of transfer, sperm donation, fertilization method, fresh/frozen embryo transfer, blastocyst/cleavage-stage transfer, assisted hatching, antral follicle count, basal serum FSH level, endometrial thickness, number of embryos transferred, and singleton or twin pregnancy). In the subgroup analysis, we explored the relationships between pre-pregnancy BMI and miscarriages in the subgroup of singleton and twin pregnancies. We conducted a sensitivity analysis using the BMI criteria proposed by the WHO. We also used restricted cubic splines to flexibly model the association between pre-pregnancy BMI and miscarriages. Statistical analyses were performed using the SAS software package (version 9.4; SAS Institute Inc., Cary, NC, USA). All P-values were two-sided with a significance level of < 0.05.

## Results

### Participants’ Characteristics

A total of 13,828 pregnant women who underwent ART and 14,994 pregnancy cycles were analyzed in the study. Pregnant women and pregnancy cycles were divided into underweight, normal weight, overweight, and obese groups according to the Chinese criteria. The patients’ mean age was 29.79 ± 4.08 years; 9,892 pregnancy cycles (65.97%) were embryos transferred between 2013 and 2015 and 10,699 (71.36%) were IVF treatment. The characteristics of participants are presented in **Table 1**. The overweight and obese groups were older and more likely to have higher gravidity and parity, higher antral follicle count, greater endometrial thickness, ovarian cause of infertility, and blastocyst transfer. Finally, the overweight and obese groups were more likely to have lower FSH levels, less sperm donation, and less ICSI treatment.

**Table 1:** Patient and cycle characteristics in ART pregnancies.

	Underweight	Normal weight	Overweight	Obese	*χ^2^/F* value	*P* value
**Patient characteristics**						
Patient number	1304	9409	2571	544		
Frequency of ART pregnancy, n (%)						
1	1211 (92.87)	8646 (91.89)	2370 (92.18)	493 (90.63)	2.994	0.393
≥ 2	93 (7.13)	763 (8.11)	201 (7.82)	51 (9.38)		
Age (year), mean ± SD	28.63 ± 3.73	29.76 ± 4.03	30.35 ± 4.25	30.42 ± 4.18	161.362	<0.001^a^
Smoking history, n (%)						
Yes	2 (0.15)	30 (0.32)	10 (0.39)	5 (0.92)	6.051	0.091^b^
No	1302 (99.85)	9379 (99.68)	2561 (99.61)	539 (99.08)		
Gravidity, n (%)						
0	863 (66.18)	5485 (58.30)	1433 (55.74)	314 (57.72)		
1-2	376 (28.83)	3202 (34.03)	902 (35.08)	190 (34.93)	47.656	<0.001
≥3	65 (4.98)	722 (7.67)	236 (9.18)	40 (7.35)		
Parity, n (%)						
0	1234 (94.63)	8599 (91.39)	2268 (88.21)	488 (89.71)	48.393	<0.001
≥1	70 (5.37)	810 (8.61)	303 (11.79)	56 (10.29)		
Main etiology of infertility, n (%)						
Tubal factor	566 (43.40)	4576 (48.53)	1252 (48.70)	235 (43.20)	169.889	<0.001
Ovarian factor	40 (3.07)	369 (3.92)	209 (8.13)	64 (11.76)		
Male factor	285 (21.86)	1875 (19.93)	419 (16.30)	97 (17.83)		
Other reasons	413 (31.67)	2589 (27.52)	691 (26.88)	148 (27.21)		
**Pregnancy cycle characteristics**						
Number of ART pregnancy cycles	1397	10196	2804	597		
Year of transfer, n (%)						
2006–2009	131 (9.34)	813 (7.94)	150 (5.35)	25 (4.19)		
2010–2012	375 (26.84)	2804 (27.50)	685 (24.43)	119 (19.93)	74.699	<0.001
2013–2015	891 (63.78)	6579 (64.53)	1969 (70.22)	453 (75.88)		
Sperm donation						
Yes	134 (9.59)	672 (6.59)	138 (4.92)	24 (4.02)	39.854	<0.001
No	1263 (90.41)	9524 (93.41)	2666 (95.08)	573 (95.98)		
Fertilization method, n (%)						
ICSI	429 (30.71)	2706 (26.54)	675 (24.07)	140 (23.45)		
IVF	945 (67.64)	7262 (71.22)	2054 (73.25)	438 (73.47)	28.618	<0.001
IVF + ICSI	23 (1.65)	228 (2.24)	75 (2.67)	19 (3.18)		
Timing of embryo transfer, n (%)						
Fresh embryo transfer	790 (56.55)	5914 (58.00)	1588 (56.63)	339 (56.78)	2.536	0.469
Frozen embryo transfer	607 (43.45)	4282 (42.00)	1216 (43.37)	258 (43.22)		
Day 3 or 5, n (%)						
Cleavage stage transfer	925 (66.21)	6730 (66.01)	1731 (61.73)	351 (58.79)	28.655	<0.001
Blastocyst transfer	472 (33.79)	3466 (33.99)	1073 (38.27)	246 (41.21)		
Assisted hatching, n (%)						
Yes	381 (27.27)	2902 (28.46)	809 (28.85)	158 (26.47)	2.252	0.522
No	1016 (72.73)	7294 (74.54)	1995 (71.15)	439 (73.53)		
Antral follicle count, Median (IQR)	12 (9, 16)	12 (9, 16)	13 (10, 18)	14 (10, 20)	81.749	<0.001^a^
Basal serum FSH level (U/L), Median (IQR)	7.05 (5.99, 8.18)	6.54 (5.58, 7.75)	6.31 (5.36, 7.35)	6.06 (5.20, 7.07)	216.551	<0.001^a^
Endometrial thickness (mm), median (IQR)	10.40 (9.20, 11.80)	10.50 (9.20, 12.00)	10.50 (9.20, 12.10)	10.80 (9.40, 12.20)	13.713	0.003^a^
No. of embryos transferred, n (%)						
1	230 (16.46)	1585 (15.54)	501 (17.87)	94 (15.75)		
2	1064 (76.16)	7769 (76.20)	2076 (74.04)	468 (78.39)	14.357	0.026
≥3	103 (7.37)	842 (8.26)	227 (8.10)	35 (5.86)		
Singleton or twin pregnancy, n (%)						
singleton	950 (68.00)	7003 (68.68)	1966 (70.11)	409 (68.51)	2.715	0.438
twin ^c^	447 (32.00)	3193 (31.32)	838 (29.86)	188 (31.49)		

^a^Kruskal-Wallis test; ^b^Fisher exact test; ^c^Twin pregnancy included 93 triplet pregnancies.

IQR, interquartile range.

### BMI and Early Miscarriage

Overall, the rate of early miscarriage was 11.87% among all pregnancy cycles. The rates of early miscarriage varied according to pre-pregnancy BMI. Among the underweight, normal weight, overweight, and obese groups, the rates of early miscarriage were 10.67%, 11.44%, 13.20%, and 15.91% (P < 0.001), respectively (see **Table 2** for details).

**Table 2:** Relationship between miscarriage and pre-pregnancy BMI in ART pregnancies.

Pregnancy outcomes	Total (n = 14994)	Underweight (n=1397)	Normal weight (n=10196)	Overweight (n=2804)	Obese (n=597)	*χ^2^* value	*P* value
Early miscarriage (<12 weeks), n (%)	1780 (11.87)	149 (10.67)	1166 (11.44)	370 (13.20)	95 (15.91)	17.809	< 0.001
Miscarriage (<20 weeks), n (%)	1932 (12.89)	155 (11.10)	1269 (12.45)	401 (14.30)	107 (17.92)	24.244	< 0.001

After adjusting for baseline covariates (age, smoking history, gravidity, parity, etiology of infertility, and year of transfer), the obesity group had a 32% increased risk of early miscarriage relative to the normal weight group (relative risk [RR] = 1.32, 95% confidence interval [CI]: 1.08–1.61). After adjusting for all baseline covariates, this association remained statistically significant (RR = 1.36, 95% CI: 1.12–1.65). Additionally, after adjusting for all baseline covariates, for a one-unit increment of pre-pregnancy BMI, the RR of early miscarriage increased by 3% (RR = 1.03, 95% CI: 1.01–1.04) (see **Table 3** for details).

**Table 3:** Effects of pre-pregnancy BMI on miscarriage: results from the GEE models analysis.

Pregnancy outcomes	Model 1	Model 2	Model 3
	Crude RR (95% CI), *P* value	Adjusted RR (95% CI), *P* value	Adjusted RR (95% CI), *P* value
Early miscarriage			
Underweight	0.93 (0.79–1.09), 0.394	1.00 (0.85–1.17), 0.975	0.99 (0.84, 1.16), 0.877
Normal weight	Ref	Ref	Ref
Overweight	1.15 (1.03–1.29), 0.013	1.11 (0.99–1.24), 0.072	1.11 (0.99, 1.24), 0.064
Obese	1.39 (1.14–1.70), 0.001	1.32 (1.08–1.61), 0.006	1.36 (1.12, 1.65), 0.002
Early miscarriage			
BMI	1.03 (1.02–1.05), < 0.001	1.02 (1.01–1.04), 0.001	1.03 (1.01, 1.04), < 0.001
Miscarriage			
Underweight	0.89 (0.76–1.04), 0.149	0.95 (0.81–1.11), 0.538	0.94 (0.81, 1.10), 0.466
Normal weight	Ref	Ref	
Overweight	1.15 (1.03–1.28), 0.011	1.10 (0.99–1.23), 0.075	1.10 (0.99, 1.22), 0.071
Obese	1.44 (1.20–1.73), < 0.001	1.36 (1.13–1.64), 0.001	1.40 (1.17, 1.68), < 0.001
Miscarriage			
BMI	1.04 (1.02–1.05), < 0.001	1.03 (1.01–1.04), < 0.001	1.03 (1.02, 1.04), < 0.001

Model 2 adjusted age, smoking history, gravidity, parity, etiology of infertility, year of transfer.

Model 3 adjusted all baseline covariates (age, smoking history, gravidity, parity, etiology of infertility, year of transfer, sperm donation, fertilization method, frozen or fresh embryo transfer, cleavage stage or blastocyst transfer, assisted hatching, antral follicle count, basal serum FSH, endometrial thickness, no. of embryos transferred, and singleton or twin pregnancy).

### BMI and Miscarriage

Overall, the rate of miscarriage was 12.89% among all pregnancy cycles. The rates of miscarriage varied according to pre-pregnancy BMI. Among the underweight, normal weight, overweight, and obese groups, the rates of miscarriage were 11.10%, 12.45%, 14.30%, and 17.92% (P < 0.001), respectively (see **Table 2** for details).

After adjusting for baseline covariates (age, smoking history, gravidity, parity, and etiology of infertility, year of transfer), the obesity group had a 36% increased risk of miscarriage, compared with the normal weight group (RR = 1.36, 95% CI: 1.13–1.64). After adjusting for all baseline covariates, this association remained statistically significant (RR = 1.40, 95% CI: 1.17–1.68). Additionally, after adjusting for all baseline covariates, with a one-unit increment of pre-pregnancy BMI, the RR of miscarriage increased by 3% (RR = 1.03, 95% CI: 1.02–1.04) (see **Table 3** for details).

### Subgroup Analyses

Subgroup analyses of the relationship between pre-pregnancy BMI and miscarriage were performed by singleton and twin pregnancies (see **Table 4** for details). The RRs of early miscarriage and miscarriage were significantly elevated with increased BMI for both singleton pregnancy and twin pregnancy groups. Increased pre-pregnancy BMI was associated with higher RRs of early miscarriage and miscarriage in the twin pregnancy group than in the singleton pregnancy group [early miscarriage (singleton pregnancy: RR = 1.02, 95% CI: 1.01–1.04; twin pregnancy: RR = 1.08, 95% CI: 1.03–1.13); miscarriage (singleton pregnancy: RR = 1.02, 95% CI: 1.01–1.04; twin pregnancy: RR = 1.08, 95% CI: 1.05–1.13)]. Tests of the interaction between pre-pregnancy BMI and singleton pregnancy or twin pregnancy in early miscarriage and miscarriage were statistically significant (P = 0.017 and P = 0.003, respectively).

**Table 4:** Effects of pre-pregnancy BMI on miscarriage: results from the GEE models analysis in subgroups.

Pregnancy outcomes	Singleton pregnancy			Twin pregnancy			*P* for interaction
	Total	Events	Adjusted RR (95% CI), *P* value^a^	Total	Events	Adjusted RR (95% CI),*P* value^a^	
Early miscarriage							
Underweight	950	138 (14.53)	1.02 (0.86–1.20), 0.848	447	11 (2.46)	0.79 (0.43–1.45), 0.452	
Normal weight	7003	1060 (15.14)	Ref	3193	106 (3.32)	Ref	0.018
Overweight or obese	2375	321 (16.33)	1.08 (0.97–1.21), 0.145	1026	62 (6.04)	1.70 (1.25–2.32), < 0.001	
Early miscarriage							
BMI	10328	1601 (15.50)	1.02 (1.01–1.04), 0.007	4666	179 (3.84)	1.08 (1.03–1.13), 0.001	0.017
Miscarriage							
Underweight	950	142 (14.95)	0.99 (0.84–1.16), 0.868	447	13 (2.91)	0.68 (0.39–1.19), 0.174	
Normal weight	7003	1126 (16.08)	Ref	3193	143 (4.48)	Ref	0.005
Overweight or obese	2375	427 (17.98)	1.08 (0.97–1.19), 0.163	1026	81 (7.89)	1.68 (1.29–2.19), < 0.001	
Miscarriage							
BMI	10328	1695 (16.41)	1.02 (1.01–1.04), 0.003	4666	237 (5.08)	1.08 (1.05–1.13), < 0.001	0.003

a Model adjusted by age, smoking history, gravidity, parity, etiology of infertility, year of transfer.

Overweight or obesity was associated with higher RRs of early miscarriage and miscarriage in the twin pregnancy group (early miscarriage: RR = 1.70, 95% CI: 1.25–2.32; miscarriage: RR = 1.68, 95% CI: 1.29–2.19); however, overweight or obesity was not significantly associated with higher RRs of early miscarriage or miscarriage in the singleton pregnancy group (early miscarriage: RR = 1.08, 95% CI: 0.97–1.21; miscarriage: RR = 1.08, 95% CI: 0.97–1.19). Moreover, the tests of the interaction between the pre-pregnancy BMI and singleton pregnancy or twin pregnancy in early miscarriage and miscarriage were statistically significant (P = 0.018 and P = 0.005, respectively).

### Sensitivity Analyses

**Figure 2:**
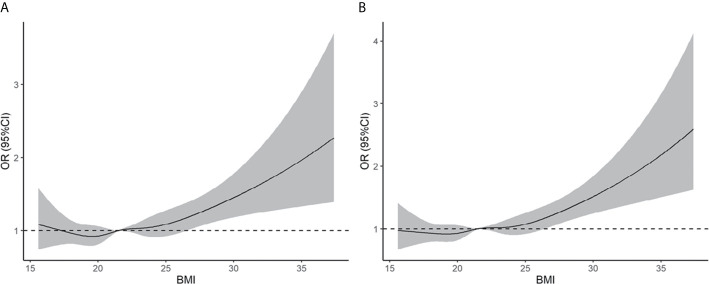
Association of pre-pregnancy BMI with early miscarriage **(A)** and miscarriage **(B)**. Estimates adjusted for age, smoking history, gravidity, parity, etiology of infertility, year of transfer, sperm donation, fertilization method, frozen or fresh embryo transfer, cleavage stage or blastocyst transfer, assisted hatching, antral follicle count, basal serum FSH, endometrial thickness, no. of embryos transferred, and singleton or twin pregnancy. OR, odds ratio.

Based on the BMI criteria proposed by the WHO, obesity was associated with an increased risk of early miscarriage and miscarriage relative to the normal weight group (early miscarriage: RR = 1.43, 95% CI: 1.09–1.90; miscarriage: RR = 1.48, 95% CI: 1.13–1.95). We also found that being overweight was associated with an increased risk of early miscarriage and miscarriage relative to the normal weight group (early miscarriage: RR = 1.19, 95% CI: 1.06–1.35; miscarriage: RR = 1.19, 95% CI: 1.06–1.34) (see **Supplementary Table 1**). As shown in **Figure 2**, the results of restricted cubic splines revealed that the risk of early miscarriage and miscarriage was relatively flat until a BMI of approximately 24 kg/m^2^ and then started to increase rapidly afterward.

## Discussion

In a large cohort of pregnant women undergoing ART treatment, we found that pre-pregnancy obesity was associated with a higher risk of early miscarriage and miscarriage, whereas pre-pregnancy underweight was not associated with an increased risk of early miscarriage and miscarriage. Additionally, we found interactions between pre-pregnancy BMI and singleton or twin pregnancy in the risk of early miscarriage and miscarriage. Twin pregnancy increased the RR of increased pre-pregnancy BMI in early miscarriage and miscarriage.

In our study, the obese group had 36% and 40% increased risk of early miscarriage and miscarriage, respectively, compared with the normal weight group. In spontaneous pregnancies, pre-pregnancy obesity is a known risk factor for pregnancy complications and adverse perinatal outcomes (24–26). Balsell et al. conducted a meta-analysis that included 32 studies with a total of 265,760 women, and reported that pre-pregnancy obesity increased the risk of miscarriage in subgroups of the cohort study and case control study (RR = 1.21, 95% CI: 1.15–1.27; odds ratio [OR] = 1.26, 95% CI: 1.01–1.57) (16). In related ART studies, Kawwass et al. confirmed that pre-pregnancy obesity was associated with increased risk of miscarriage (RR = 1.23, 95% CI: 1.20–1.26) (27). Additionally, Metwally et al. conducted a meta-analysis that included 16 studies with more than 16,000 participants, and the results revealed that BMI ≥ 25 kg/m^2^ increased the risk of miscarriage (OR = 1.67, 95% CI: 1.25–2.25), and overweight or obesity was associated with a higher risk of miscarriage in the oocyte donation and ovulation induction groups (OR = 1.52, 95% CI: 1.10–2.09; OR = 5.11, 95% CI: 1.76–14.83); this trend was also seen in the IVF/ICSI group, but the association was not statistically significant (OR = 1.52, 95% CI: 0.88–2.61) (28).

Several mechanisms have been proposed to explain the association between pre-pregnancy obesity and miscarriage. First, the association between increased BMI and increased risk of miscarriage might be related to the action of leptin produced in the adipose tissue (29, 30). Leptin receptors are expressed in the secretory endometrium and regulate endometrial angiogenesis (31), and thus may influence implantation (29, 30). Second, a high BMI has been confirmed to be associated with insulin resistance. Insulin resistance increases the risk of miscarriage both after natural conception (32) and and ART treatment (33). Insulin resistance involved in miscarriage is related to diminished endometrial production of adhesion factors, such as insulin-like growth factor (34–36). Additionally, obesity is associated with reduction in serum progesterone level in early pregnancy (37–39), which is essential for maintaining pregnancy and predicting subsequent pregnancy loss (40).

In our study, compared with women with pre-pregnancy normal weight, women with pre-pregnancy underweight had a similar chance of miscarriage after ART treatment. A large sample of 180,855 ART pregnancies in the USA also confirmed that underweight was not significantly associated with increased miscarriage (RR = 1.04, 95% CI: 0.98–1.11) (27). However, other studies have drawn contrasting conclusions. Veleva et al. used 3,330 first pregnancy cycles conceived through IVF/ICSI and frozen-thawed embryo transfer, and Cai et al. used 4,798 first fresh transfer cycles, conceived through IVF, to explore the association between low BMI and miscarriage, and reported that low BMI was associated with an increased risk of miscarriage in ART treatment (19, 20). The inconsistent conclusions might be due to the relatively small sample size of the underweight group in Veleva et al.’s and Cai et al.’s studies, which might lack sufficient statistical power. Additionally, Veleva et al. and Cai et al. only analyzed the first pregnancy cycle, while our study used all pregnancy cycles to explore the association between BMI and miscarriages. In our subgroup analysis and sensitivity analysis, pre-pregnancy underweight was not associated with an increased risk of early miscarriage and miscarriage with ART treatment.

We found interactions between pre-pregnancy BMI and singleton pregnancy or twin pregnancy in early miscarriage and miscarriage. Although miscarriage rates were higher in the singleton pregnancy group than in the twin pregnancy group, increased BMI was associated with a higher RR of early miscarriage and miscarriage in the twin pregnancy group than in the singleton pregnancy group. In ART treatment, twin pregnancies are mainly due to the transfer of multiple embryos (two or three embryos) related to lower reproductive capability, such as advanced age, poor uterine receptivity, and low-grade embryo quality (41). Pre-pregnancy obesity has been recognized as a major risk factor for adverse maternal outcomes, including hypertensive disorders and glucose intolerance (42). Women with twins are at an increased risk for many of the complications associated with maternal obesity described above, which may be related to an increased risk of miscarriage. The results of our study demonstrated that efforts should be devoted to the management of anthropometric parameters before ART treatment to prevent adverse pregnancy outcomes.

In this study, in contrast to other large studies on the relationship between BMI and pregnancy outcomes, we found interactions between maternal BMI and singleton or twin pregnancy with respect to miscarriage in ART treatment. Moreover, we confirmed that obesity increased the risk of early miscarriage and miscarriage after ART treatment. Additionally, overweight and obesity were classified according to the Chinese standard, which is more applicable in Chinese adults than the WHO standard. This study has some limitations. First, this was an observational study in which the causality between BMI and miscarriages could not be established. Additionally, although we used multivariable regressions to control for potential confounders, the findings might be confounded by unmeasured covariates (e.g., alcohol consumption during pregnancy and maternal nutritional status) because the data in hospital information systems were limited.

## Conclusion

In conclusion, our findings indicate that pre-pregnancy obesity is associated with an increased risk of early miscarriage and miscarriage in a population that underwent ART treatment. Interactions between pre-pregnancy BMI and singleton or twin pregnancy with respect to early miscarriage and miscarriage were observed, and twin pregnancy increased the influence of increased pre-pregnancy BMI on early miscarriage and miscarriage. Women before ART treatment should maintain a normal BMI to prevent adverse pregnancy outcomes.

## Data Availability Statement

The raw data supporting the conclusions of this article will be made available by the authors, without undue reservation.

## Ethics Statement

The studies involving human participants were reviewed and approved by the human research ethics committee of the Northwest Women’s and Children’s Hospital. The ethics committee waived the requirement of written informed consent for participation.

## Author Contributions

PQ, SD, WS, JS, and CZ conceived and designed the study. PQ, MY, DZ, DW, and CZ drafted and revised the manuscript. PQ, MY, and DZ analyzed and interpreted the data. PQ, MY, and DZ collected and cleared the data. All authors contributed to the article and approved the submitted version.

## Funding

This work was financially supported by “the Fundamental Research Funds for the Central Universities” (China) (no. xzy012019116), the Key Research and Development Program of Shaanxi Province (no. 2020SF-031), and the National Natural Science Foundation of China (no. 81771657).

## Conflict of Interest

The authors declare that the research was conducted in the absence of any commercial or financial relationships that could be construed as a potential conflict of interest.

The reviewer XL declared a shared affiliation with several of the authors, MY and SD, to the handling editor at time of review.
