# Supplementary material for: Association Between Pre-Pregnancy Body Mass Index and Miscarriage in an Assisted Reproductive Technology Population: A 10-Year Cohort Study
Source: Front Endocrinol (Lausanne). 2021 Jun 16;12:646162. doi: 10.3389/fendo.2021.646162 (PMC8242335; doi:10.3389/fendo.2021.646162)
Supplement: Supplementary file 1 [file Table_1.docx]

**Supplementary Table** 1. Effects of pre-pregnancy BMI on miscarriage based on the BMI criteria proposed by the WHO

| Pregnancy outcomes | Model 1 | Model 2 | Model 3 |
| --- | --- | --- | --- |
|  | Crude RR (95% CI), *P* value | Adjusted RR (95% CI), *P* value | Adjusted RR (95% CI), *P* value |
| Early miscarriage |  |  |  |
| Underweight | 0.93 (0.79, 1.09), 0.361 | 1.00 (0.85, 1.17), 0.972 | 0.99 (0.84, 1.15), 0.857 |
| Normal weight | Ref | Ref | Ref |
| Overweight | 1.24 (1.10, 1.41), < 0.001 | 1.20 (1.06, 1.35), 0.004 | 1.19 (1.06, 1.35), 0.004 |
| Obese | 1.39 (1.14, 1.70), 0.006 | 1.42 (1.07, 1.89), 0.015 | 1.43 (1.09, 1.90), 0.011 |
| Miscarriage |  |  |  |
| Underweight | 0.89 (0.76, 1.04), 0.132 | 0.95 (0.82, 1.11), 0.537 | 0.94 (0.81, 1.10), 0.452 |
| Normal weight | Ref | Ref |  |
| Overweight | 1.25 (1.11, 1.40), < 0.001 | 1.20 (1.07, 1.35), 0.003 | 1.19 (1.06, 1.34), 0.003 |
| Obese | 1.55 (1.18, 2.04), 0.002 | 1.48 (1.12, 1.94), 0.005 | 1.48 (1.13, 1.95), 0.004 |

Model 2 adjusted age, smoking history, gravidity, parity, etiology of infertility, year of transfer.

Model 3 adjusted all baseline variates (age, smoking history, gravidity, parity, etiology of infertility, year of transfer, sperm donation, fertilization method, frozen or fresh embryo transfer, cleavage stage or blastocyst transfer, assisted hatching, antral follicle count, basal serum FSH, endometrial thickness, no. of embryos transferred, and singleton or twin pregnancy).
